# Supplementary material for: Risk of miscarriage in women with chronic diseases in Norway: A registry linkage study
Source: PLoS Med. 2021 May 10;18(5):e1003603. doi: 10.1371/journal.pmed.1003603 (PMC8143388; doi:10.1371/journal.pmed.1003603)
Supplement: S1 Analysis Plan — (DOCX) [file pmed.1003603.s002.docx]

S1 Analysis plan

**Risk of miscarriage according to chronic underlying conditions**

**Objective**: Our objective was to study the risk of miscarriage in relation to mother´s chronic illness present prior to pregnancy.

**Study population:** Registered pregnancies in Norway between 2010 and 2016 identified through three national health registries (birth register, general practitioner data, and patient registries).

**Data sources:** We will link information from the birth register, general practitioner database, and the patient registry, in order to capture all registered/recognized pregnancies. This includes all live births, stillbirth, induced abortions and miscarriages. We will not be able to capture unrecognized/very early miscarriages, which do not result in contact with the health-care services.

**Exposure**: The exposure of interest is a history of different chronic conditions prior to pregnancy. The specific conditions that will be evaluated, and the administrative codes which will be used to define them, are listed in table 1. We will define the presence of these conditions as a minimum of two registrations of the administrative codes of interest, where at least one of the registration occurred prior to the estimated start of the pregnancy.

Table 1: Illustration of how the project will capture maternal chronic conditions in the health registries

| Diseases | International Classification of Diseases (ICD-10) codes | International Classification of Primary Care (ICPC-2) codes |
| --- | --- | --- |
| Type 1 diabetes | E10 | T89 |
| Celiac disease | K90.0 |  |
| Systemic lupus erythematosus | M32 |  |
| Multiple sclerosis* |  | N86 |
| Rheumatoid arthritis/ Ankylosing spondylitis | M05-M09, M45 | L88 |
| Ulcerative colitis | [K50.0](javascript:NavigateTo('icd10','ICD10SysDel',2616425)), [K50.1](javascript:NavigateTo('icd10','ICD10SysDel',2616426)), [K50.8](javascript:NavigateTo('icd10','ICD10SysDel',2616427)), [K50.9](javascript:NavigateTo('icd10','ICD10SysDel',2616428)), [K51.0](javascript:NavigateTo('icd10','ICD10SysDel',2616430)), [K51.1](javascript:NavigateTo('icd10','ICD10SysDel',2616431)), [K51.2](javascript:NavigateTo('icd10','ICD10SysDel',2616432)), [K51.3](javascript:NavigateTo('icd10','ICD10SysDel',2616433)), [K51.4](javascript:NavigateTo('icd10','ICD10SysDel',2616434)), [K51.5](javascript:NavigateTo('icd10','ICD10SysDel',2616435)), [K51.8](javascript:NavigateTo('icd10','ICD10SysDel',2616436)), [K51.9](javascript:NavigateTo('icd10','ICD10SysDel',2616437)), [K52.0](javascript:NavigateTo('icd10','ICD10SysDel',2616439)) | D94 |
| Psoriasis* |  | S91 |
| Crohn´s disease | K50 |  |
| Addison disease | E27.1, E27.2 |  |
| Haemolytic anemia | D55- D59 | B78 |
| Autoimmune thyroiditis | E06.3 |  |
| Type 2 diabetes | E11 | T90 |
| Hypertensive disorders | I10-I15 | K85-87 |
| Atherosclerosis | I25.1, I70 |  |
| Hypothyroidism | E01 E03 | T86 |
| Hyperthyroidism | E05 | T85 |
| Hypoparathyroidism | E20 |  |
| Hyperparathyroidism | E21.0 , E21.1, E21.2, E21.3 |  |
| Cushing syndrome | E24 |  |
| Epilepsy | G40-41 | N88 |
| Migraine | G43 G44.1 | N89 |
| Asthma | J45 and J46 | R96 |
| Allergic rhinitis | J30 | R97 |
| Atopic dermatitis | L20 | S87 |
| Polycystic ovary syndrome | E28.2 |  |
| Endometriosis | N80 |  |

*Information on these conditions is not available from the patient registry.

**Outcome**: The outcome of interest is miscarriage. A miscarriage in the birth registry is defined as a fetal death before 20 gestational weeks with a birthweight less than 500 grams. Miscarriages which occurred before 12 gestational weeks, and which are therefore not recorded in the birth registry, will be identified in the patient and general practitioner database using the codes defined in table 2. The comparison group will include all live births, stillbirths, and a proportion of induced abortions. The duration of pregnancies in the birth registry will be estimated as the date of birth minus the gestational age in days. The gestational age of miscarriages and induced abortions in the patient registry and the general practitioner database will be set to 12 weeks, under the assumption that they should be registered in the birth registry if the gestational length was longer than this.

Table 2: Codes that are used to identify miscarriages/induced abortion in the registries

| Pregnancy outcome | International Classification of Diseases (ICD-10) codes | International Classification of Primary Care (ICPC-2) codes |
| --- | --- | --- |
| Miscarriage | O01 Hydatidiform mole  O02.0 Blighted ovum and nonhydatidiform mole  O02.1 Missed abortion  O02.8 Other specified abnormal products of conception  O02.9 Abnormal product of conception, unspecified  O03 Spontaneous abortion  O20.0 Threatened abortion | W03 Bleeding in pregnancy  W82 Spontaneous abortion  W91 Uncomplicated delivery stillborn  W93 Complicated delivery stillborn |
| Induced abortion | O04 Medical abortion  O05 Other abortion  O06 Unspecified abortion |  |

Quality control of miscarriages and induced abortions identified in the patient registry and the general practitioner database:

We will take the following steps to ensure that we are capturing unique miscarriages and induced abortions in the patient registry and general practitioner database:

1. There should be at least 90 days between registrations of codes for miscarriage in the general practitioner database to be counted as a new pregnancy, while there should be at least 42 days between registrations of codes used to defined miscarriage and induced abortion in the patient registry.
2. Registrations in the patient registry and general practitioner database should not have occurred within the estimated duration of a registered pregnancy in the birth registry. The duration of pregnancies in the birth registry will be estimated as the date of birth minus the gestational age in days.
3. Any registrations of miscarriage or induced abortions should be at least 90 days (general practitioner database) or 42 days (patient registry) after a registered delivery in the birth registry to be counted as a new pregnancy.

**Statistical analysis:** We will estimate the associations using log-binomial regression, estimating relative risks and 95% confidence intervals.

To obtain estimates of the associations accounting for induced abortions, we will randomly sampled 20% of the induced abortions a total of 1,000 times and calculated the effect estimates as an average across these estimates. The standard errors of the effect estimates will be estimated by combining the estimated variance of the betas across and between the iterations using the following equation drawing on Rubin’s rules:

$$\sigma^{2}= \bar{U}+\left\{ 1+\frac{1}{m} \right\} B$$

, where $\bar{U}$ is the estimate of the variance of the beta coefficient within the iteration (calculated as the squared of the standard error), and $B$ is the estimate of the variance of the beta coefficient between the iterations.
