# Supplementary material for: Risk of miscarriage in women with chronic diseases in Norway: A registry linkage study
Source: PLoS Med. 2021 May 10;18(5):e1003603. doi: 10.1371/journal.pmed.1003603 (PMC8143388; doi:10.1371/journal.pmed.1003603)
Supplement: S1 Text — (DOCX) [file pmed.1003603.s003.docx]

**S1 Text. Supplementary Methods**

**Details of data sources and linkages**

All data in Norwegian national health registries are registered with the personal identification number (pin), which is given all citizens in Norway for identification and public administration purposes. This pin enables linkages of individual level information across registries.

*The Medical Birth Registry of Norway (MBRN)*

The Norwegian national birth registry includes information on all pregnancies ending in gestational 12 or later from 1967 onwards. The registry is registered with the woman’s pin includes information on live births, stillbirths, late miscarriages and late induced abortions. Midwives register information on maternal background characteristics and health during pregnancy, in addition to information on pregnancy outcomes and neonatal health of offspring.

*The Norwegian Patient Registry (NPR)*

The Norwegian patient registry includes individual level information on all contacts with specialist health-care services since 2008. Information registered includes the pin, admission and discharge dates, the type of department and level of care received, in addition to main and secondary discharge codes. The discharge codes are coded according to the International Classification of Diseases version 10. We used the discharge codes to identify primary and secondary codes indicating the presence of a miscarriage. With cross-referencing against the birth registry, we used this registry to identify miscarriages and induced abortions that occurred before 12 completed gestational weeks.

### *[Norway Control and Payment of Health Reimbursement (KUHR)](http://ghdx.healthdata.org/series/norway-control-and-payment-health-reimbursement-kuhr-database)*

The KUHR database includes individual level information on all contact with primary health-care services since 2006 onwards. Information registered includes the pin, the date, the reimbursement code for payment of services, and the main and secondary medical diagnoses. The diagnoses are coded according to the International Classification of Primary Care version 2. We used the medical codes in the KUHR database to identify all miscarriages occurring before 12 gestational weeks that were not identified in the birth or patient registries. This subgroup of miscarriages therefore reflect those only seen by a general practitioner and not referred for any follow up in the specialist health-care services.

**Estimation of the proportion of induced abortions for adjustment of miscarriage risk**

In 1983, Susser proposed adding 50% of induced abortions to the denominator, assuming that the gestational-week distribution of induced abortions and miscarriages are roughly similar [1]. However, based on data from the Norwegian anonymous induced abortion register [2], induced abortions in Norway, occur relatively early compared with miscarriages, so that adding 50% of induced abortions would be an over-adjustment.

A formal solution would be a life-table analysis of competing risks, which would require information on gestational-week-specific risks for both induced abortion and miscarriage. In the Norwegian data sets, we have no information on week-specific risk of miscarriage, and information on week-specific risk of induced abortion is available only for the whole population, not within disease-specific strata. In order to provide a rough adjustment for induced abortion appropriate to our data, we identified a referent set of week-specific miscarriage risks [3], and combined this with the overall week-specific risk of induced abortions from the anonymous induced abortion register. With these two data sets, we could generate the estimated number of miscarriages that occurred among pregnancies intended for termination, and the total number of miscarriages that could have occurred in those pregnancies if no termination had occurred. The ratio of these two numbers was 20%, which serves as a rough estimate of the proportion of induced abortions needed to add to the denominator of miscarriage risk to minimize bias.

This adjustment is subject to at least two important caveats. We must assume that the published set of referent gestational-week-specific miscarriage risks provide a reasonable estimate of the risk in Norway, and that the overall gestational-week distribution of induced abortions in Norway is similar within each category of chronic disease.

To obtain estimates of the associations accounting for induced abortions, we randomly sampled 20% of the induced abortions a total of 1,000 times and calculated the effect estimates as an average across these estimates. The standard errors of the effect estimates were estimated by combining the estimated variance of the betas across and between the iterations using the following equation drawing on Rubin’s rules:

$$\sigma^{2}= \bar{U}+\left\{ 1+\frac{1}{m} \right\} B$$

, where $\bar{U}$ is the estimate of the variance of the beta coefficient within the iteration (calculated as the squared of the standard error), and $B$ is the estimate of the variance of the beta coefficient between the iterations.

References

1. Susser E. Spontaneous abortion and induced abortion: an adjustment for the presence of induced abortion when estimating the rate of spontaneous abortion from cross-sectional studies. *Am J Epidemiol.* 1983; 117:305-8.

2. Norwegian Institute of Public Health. Norwegian Registry of Pregnancy Termination [<https://www.fhi.no/en/hn/health-registries/registry-of-pregnancy-termination/>] Accessed 01.06.2019.

3. Goldhaber MK, Fireman BH. The fetal life table revisited: spontaneous abortion rates in three Kaiser Permanente cohorts. *Epidemiology.* 1991; 2:33-9.
