# Supplementary material for: Risk of miscarriage in women with chronic diseases in Norway: A registry linkage study
Source: PLoS Med. 2021 May 10;18(5):e1003603. doi: 10.1371/journal.pmed.1003603 (PMC8143388; doi:10.1371/journal.pmed.1003603)
Supplement: S1 Table — (DOCX) [file pmed.1003603.s004.docx]

S1 Table. Diagnostic codes used to define chronic diseases in specialist care (ICD-10 codes), and in primary care (ICPC- codes).

| Group of diseases | Diseases | International Classification of Diseases (ICD-10) codes | International Classification of Primary Care (ICPC-2) codes |
| --- | --- | --- | --- |
| Autoimmune diseases | Type 1 diabetes | E10 | T89 |
|  | Celiac disease | K90.0 |  |
|  | Systemic lupus erythematosus | M32 |  |
|  | Multiple sclerosis* |  | N86 |
|  | Rheumatoid arthritis/ Ankylosing spondylitis | M05-M09, M45 | L88 |
|  | Ulcerative colitis | [K50.0](javascript:NavigateTo('icd10','ICD10SysDel',2616425)), [K50.1](javascript:NavigateTo('icd10','ICD10SysDel',2616426)), [K50.8](javascript:NavigateTo('icd10','ICD10SysDel',2616427)), [K50.9](javascript:NavigateTo('icd10','ICD10SysDel',2616428)), [K51.0](javascript:NavigateTo('icd10','ICD10SysDel',2616430)), [K51.1](javascript:NavigateTo('icd10','ICD10SysDel',2616431)), [K51.2](javascript:NavigateTo('icd10','ICD10SysDel',2616432)), [K51.3](javascript:NavigateTo('icd10','ICD10SysDel',2616433)), [K51.4](javascript:NavigateTo('icd10','ICD10SysDel',2616434)), [K51.5](javascript:NavigateTo('icd10','ICD10SysDel',2616435)), [K51.8](javascript:NavigateTo('icd10','ICD10SysDel',2616436)), [K51.9](javascript:NavigateTo('icd10','ICD10SysDel',2616437)), [K52.0](javascript:NavigateTo('icd10','ICD10SysDel',2616439)) | D94 |
|  | Psoriasis* |  | S91 |
|  | Crohn´s disease | K50 |  |
|  | Addison disease | E27.1, E27.2 |  |
|  | Haemolytic anemia | D55- D59 | B78 |
|  | Autoimmune thyroiditis | E06.3 |  |
| Cardiometabolic diseases | Type 2 diabetes | E11 | T90 |
|  | Hypertensive disorders | I10-I15 | K85-87 |
|  | Atherosclerosis | I25.1, I70 |  |
| Endocrinological diseases | Hypothyroidism | E01 E03 | T86 |
|  | Hyperthyroidism | E05 | T85 |
|  | Hypoparathyroidism | E20 |  |
|  | Hyperparathyroidism | E21.0 , E21.1, E21.2, E21.3 |  |
|  | Cushing syndrome | E24 |  |
| Neurological diseases | Epilepsy | G40-41 | N88 |
|  | Migraine | G43 G44.1 | N89 |
| Allergic diseases | Asthma | J45 and J46 | R96 |
|  | Allergic rhinitis | J30 | R97 |
|  | Atopic dermatitis | L20 | S87 |
| Reproductive diseases | Polycystic ovary syndrome | E28.2 |  |
|  | Endometriosis | N80 |  |

*Information on these conditions were not available from the patient registry.
