# Supplementary material for: Risk of miscarriage in women with chronic diseases in Norway: A registry linkage study
Source: PLoS Med. 2021 May 10;18(5):e1003603. doi: 10.1371/journal.pmed.1003603 (PMC8143388; doi:10.1371/journal.pmed.1003603)
Supplement: S2 Table — (DOCX) [file pmed.1003603.s005.docx]

S2 Table. Prevalence of pre-existing chronic diseases prior to pregnancy within miscarriages identified in the specialist and primary health-care services.

| Group of diseases | Diseases | Miscarriages identified in the birth registry and specialist care  (n=62,974) | | Miscarriages identified in the general practitioner database  (n=22,702) | |
| --- | --- | --- | --- | --- | --- |
|  |  | No. pregnancies | % | No. pregnancies | % |
| Autoimmune diseases | Type 1 diabetes | 238 | 0.38 | 51 | 0.22 |
|  | Celiac disease | 94 | 0.15 | 36 | 0.16 |
|  | Systemic lupus erythematosus | 39 | 0.06 | 4 | 0.02 |
|  | Multiple sclerosis | 92 | 0.15 | 26 | 0.11 |
|  | Rheumatoid arthritis/ Ankylosing spondylitis | 323 | 0.51 | 95 | 0.42 |
|  | Ulcerative colitis | 358 | 0.57 | 98 | 0.43 |
|  | Psoriasis | 323 | 0.51 | 138 | 0.61 |
|  | Crohn´s disease | 226 | 0.36 | 63 | 0.28 |
|  | Addison disease | 8 | 0.01 | 3 | 0.01 |
|  | Haemolytic anemia | 24 | 0.04 | 10 | 0.04 |
|  | Autoimmune thyroiditis | 53 | 0.09 | 25 | 0.11 |
| Cardiometabolic diseases | Type 2 diabetes | 342 | 0.54 | 86 | 0.38 |
|  | Hypertensive disorders | 840 | 1.33 | 245 | 1.08 |
|  | Atherosclerosis | 18 | 0.03 | 3 | 0.01 |
| Endocrinological diseases | Hypothyroidism | 1066 | 1.69 | 328 | 1.44 |
|  | Hyperthyroidism | 288 | 0.46 | 90 | 0.40 |
|  | Hypoparathyroidism | 8 | 0.01 | 1 | 0.004 |
|  | Hyperparathyroidism | 18 | 0.03 | 4 | 0.02 |
|  | Cushing syndrome | 6 | 0.01 | 4 | 0.02 |
| Neurological diseases | Epilepsy | 280 | 0.44 | 95 | 0.42 |
|  | Migraine | 2153 | 3.42 | 698 | 3.07 |
| Allergic diseases | Asthma | 1777 | 2.82 | 628 | 2.77 |
|  | Allergic rhinitis | 2472 | 3.93 | 921 | 4.06 |
|  | Atopic dermatitis | 745 | 1.18 | 275 | 1.21 |
| Reproductive diseases | Polycystic ovary syndrome | 15 | 0.02 | 5 | 0.02 |
|  | Endometriosis | 804 | 1.28 | 207 | 0.91 |
