# Supplementary material for: Risk of miscarriage in women with chronic diseases in Norway: A registry linkage study
Source: PLoS Med. 2021 May 10;18(5):e1003603. doi: 10.1371/journal.pmed.1003603 (PMC8143388; doi:10.1371/journal.pmed.1003603)
Supplement: S1 Fig — (DOCX) [file pmed.1003603.s006.docx]

S1 Fig. Unadjusted odds ratios of miscarriage according to the presence of chronic conditions prior to pregnancy
