# Supplementary material for: Risk of miscarriage in women with chronic diseases in Norway: A registry linkage study
Source: PLoS Med. 2021 May 10;18(5):e1003603. doi: 10.1371/journal.pmed.1003603 (PMC8143388; doi:10.1371/journal.pmed.1003603)
Supplement: S2 Fig — (DOCX) [file pmed.1003603.s007.docx]

S2 Fig. Adjusted * odds ratios of miscarriage according to the presence of chronic conditions prior to pregnancy excluding molar pregnancies

*Adjusted for the woman’s age at the start of pregnancy as a linear and a squared term.
