# Supplementary material for: Risk of miscarriage in women with chronic diseases in Norway: A registry linkage study
Source: PLoS Med. 2021 May 10;18(5):e1003603. doi: 10.1371/journal.pmed.1003603 (PMC8143388; doi:10.1371/journal.pmed.1003603)
Supplement: S3 Fig — (DOCX) [file pmed.1003603.s008.docx]

S3 Fig. Adjusted * odds ratios of miscarriage according to the presence of chronic conditions prior to pregnancy stratified by whether the mother was younger than 35 years (n=481,579) or 35 years or higher (n=111,430).

^*^Adjusted for the woman’s age at the start of pregnancy as a linear and a squared term.
