# Supplementary material for: Risk of miscarriage in women with chronic diseases in Norway: A registry linkage study
Source: PLoS Med. 2021 May 10;18(5):e1003603. doi: 10.1371/journal.pmed.1003603 (PMC8143388; doi:10.1371/journal.pmed.1003603)

S4 Fig. Age-associated risk of miscarriage identified in specialist (n = 62,974 pregnancies) or primary (n= 22,702 pregnancies) health-care services.


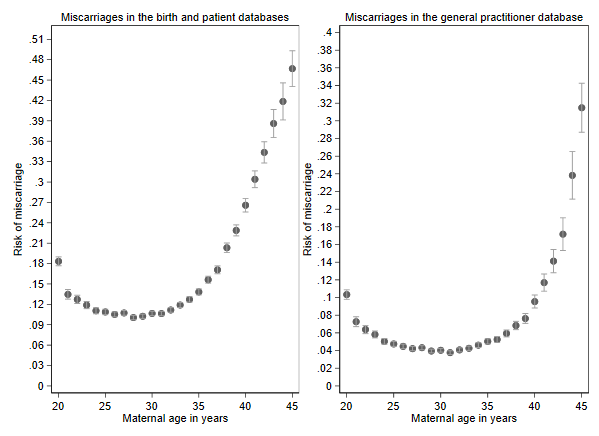

Supplement: S4 Fig — (DOCX) [file pmed.1003603.s009.docx]
