# Supplementary material for: Risk of miscarriage in women with chronic diseases in Norway: A registry linkage study
Source: PLoS Med. 2021 May 10;18(5):e1003603. doi: 10.1371/journal.pmed.1003603 (PMC8143388; doi:10.1371/journal.pmed.1003603)
Supplement: S5 Fig — (DOCX) [file pmed.1003603.s010.docx]

S5 Fig. Adjusted* odds ratios of miscarriage according to the presence of chronic conditions prior to pregnancy according to whether the miscarriage was identified in the specialist (n = 62,974 pregnancies) or primary (n= 22,702 pregnancies) health-care services.

*Adjusted for the woman’s age at the start of pregnancy as a linear and a squared term.
