# Supplementary material for: Risk of miscarriage in women with chronic diseases in Norway: A registry linkage study
Source: PLoS Med. 2021 May 10;18(5):e1003603. doi: 10.1371/journal.pmed.1003603 (PMC8143388; doi:10.1371/journal.pmed.1003603)
Supplement: Attachment — Submitted filename: 20201213_Response_letter.docx [file pmed.1003603.s012.docx]

Artur Arikainen, Phd

Associate Editor

PLOS Medicine

Dear editor,

We are grateful for the opportunity to revise our manuscript for further consideration for publication in PLOS Medicine. Please see a response to the reviewers´ comments below. We hope that you will find the manuscript improved and acceptable for publication.

Sincerely,

Maria C. Magnus (on behalf of the authors)

Researcher

Centre for Fertility and Health

Norwegian Institute of Public Health

P.O. Box 222 Skøyen

0213 Oslo

Norway

Requests from the editors:

**Comment 1: *Please revise your title according to PLOS Medicine's style - we'd suggest this includes an indication of the study design (eg, "registry linkage study) in the subtitle (ie, after a colon).**

Response comment 1: We have revised the title as requested.

New title

Risk of miscarriage in women with chronic diseases: a Norwegian registry linkage study

**Comment 2: *In the paper the authors define the study as a prospective-registry-based study, however given the analysis uses routinely collected data (the medical birth registry, the Norwegian Patient Registry, and the general practitioner database), presumably the data existed in those registries before the plan to draw them together and analyse them for the purposes of this question, was developed? In which case it might be better to define it as retrospective (in the sense the data existed before the analysis plan)? More clarity on this would be helpful.**

Response comment 2: The information in the registries is recorded prospectively. This is why we initially defined the study design as a prospective-registry-based study. We acknowledge the fact that the information in the registries was not recorded for the specific purpose of the research question addressed in this paper. In response to this comment, we have revised the description of the study design as a retrospective-registry-based study.

Abstract (p. 2):

“We conducted a retrospective registry-based study. Registered pregnancies (n=593,009) in Norway between 2010 and 2016 were identified through three national health registries (birth register, general practitioner data, and patient registries).”

Methods (p. 6):

“We conducted a retrospective registry-based study using Norwegian national registries.”

**Comment 3: *We'd suggest using the RECORD guideline (developed to help reporting of analyses conducted using registry linkage/routinely collected data) -** [**https://www.equator-network.org/reporting-guidelines/record/**](https://www.equator-network.org/reporting-guidelines/record/)**. In this case please include the completed RECORD checklist as Supporting Information. Please add the following statement, or similar, to the Methods: "This study is reported as per the RECORD guideline (S1 Checklist)". When completing the checklist, please use section and paragraph numbers, rather than page numbers.**

Response comment 3: We have incorporated the checklist as requested.

**Comment 4: : *In the last sentence of the Abstract Methods and Findings section, please include a brief note about any key limitation(s) of the study's methodology. Specifically, a reviewer notes that there are major limitations in terms of the ability to adjust for possible (likely) confounders in this study, and this certainly should be mentioned.**

Response comment 4: We have included the following sentence in the conclusion as requested.

Abstract (p. 2):

“We were not able to adjust for measures of socio-economic position of lifestyle characteristics, and unmeasured confounding might therefore influence the results. ”

**Comment 5: *Can the authors clarify at what point the analytical plan used in this paper was developed - please state this (either way) early in the Methods section.**

**a) If a prospective analysis plan (from your funding proposal, IRB or other ethics committee submission, study protocol, or other planning document written before analyzing the data) was used in designing the study, please include the relevant prospectively written document with your revised manuscript as a Supporting Information file to be published alongside your study, and cite it in the Methods section. A legend for this file should be included at the end of your manuscript.**

**b) If no such document exists, please make sure that the Methods section transparently describes when analyses were planned, and when/why any data-driven changes to analyses took place.**

**c) In either case, changes in the analysis-- including those made in response to peer review comments-- should be identified as such in the Methods section of the paper, with rationale.**

Response comment 5: We have included the following sentences in the methods as requested.

Methods (p. 8-9):

“We included a broad range of chronic diseases. We decided a priori what chronic diseases to include as exposures. No formal analysis plan was developed. The only notable changes based on what was decided a priori was the inclusion of an analysis of any cardiometabolic, any autoimmune, any reproductive, any endocrinological, any allergic, and any neurological diseases. In addition, autoimmune thyroiditis was included as part of review process.”

-----------------------------------------------------------

Comments from the reviewers:

**Reviewer #1: I confine my remarks to statistical aspects of this paper. These were well done.**

**Comment 1: My only concern is about confidence intervals (I am glad the authors did not include p values). But, since the authors have the entire population, what are these CIs? Some people posit a "super-population" of some sort. I'm not a big fan of this, pregnancies in Norway are not a random sample of pregnancies world wide (for one thing, health care is much better there than in almost any other country). But this needs to be addressed.**

**I'd be fine with no CIs, but others might object.**

**Peter Flom**

Response comment 1: We appreciate the reviewer´s perspective. Despite this being a registry-based study, there is a question about the generalizability of our findings to other populations, in addition to model uncertainty which is always present, which warrants the reporting of confidence intervals. We have included the following sentence in the discussion.

Discussion (p. 15):

“Our findings might also not be generalizable to populations with different ethnic compositions or populations without universal health-care services. ”

-----------------------------------------------------------

**Reviewer #2: This is a fairly straightforward analysis of whether chronic disease is associated miscarriage. Strengths of the paper are the large, population-based dataset, consistent coding, and careful consideration of analytic issues, such as competing risk due to induced abortion.**

**Comment 1: The authors discuss the possibility of missing diagnosis of chronic disease, but have less to say about missing diagnosis of miscarriage. Many early miscarriages are not clinically treated, many are probably not reported, and others are not even noticed. The authors discuss the overall incidence and how it compares to other populations, but not this issue. I also don't see discussion of the fact that women under treatment for some conditions may also be more aware of their bodies, or more likely to report to doctors if they do miscarry, particularly for a condition like endometriosis. An additional possible issue is competing risk from subfertility, which might be a cause for the lack of relationship with some outcomes (it is possible that some of the medications used to treat epilepsy could cause very early, i.e. undetectable, losses or infertility.)**

Response comment 1: Thank you for highlighting this important limitation. We have included the following sentences in the discussion.

Discussion (p. 15):

“We were able to identify only miscarriages that resulted in contact with health-care services (primary or secondary health care services). Very early miscarriages where the woman did not know she was pregnant or did not choose to seek medical attention could not be included.”

**Comment 2: The authors do consider the possibility of confounding, which they are able to address only to a limited extent. I would like to see this emphasized a bit more in the abstract.**

Response comment 2: We have incorporated the following sentence into the abstract.

Abstract (p. 2):

“We were not able to adjust for measures of socio-economic position or lifestyle characteristics, and unmeasured confounding might influence the results.”

-----------------------------------------------------------

**Reviewer #3: This study reports on the prospective risk of miscarriage in women with a preexisting chronic disease based on 6 years of all reported pregnancies in Norway (2010-2016). The study design ensures that the chronic disease is diagnosed prior to the pregnancy loss. The authors also undertake the task of estimating how many of the terminated pregnancies would have ended as a pregnancy loss if not the termination had been undertaken. The study is larger than previous studies, however no new information is given by the study.**

**Major issues.**

**Comment 1: Terminology: Miscarriage is per definition a loss of a confirmed intrauterine pregnancy. This requires that the pregnancy has been scanned, identification of villi or fetus in the expelled pregnancy or in the evacuate. The authors use miscarriage despite several of their included pregnancies are not confirmed intrauterine - please instead use pregnancy loss.**

Response comment 1:

**Comment 2: Definition: It is unclear why the authors included molar pregnancies in their definition of miscarriage. Please explain.**

Response comment 2: We have conducted a sensitivity analysis excluding the small number of molar pregnancies included in the definition of miscarriage (n=2367; 2.8% of all miscarriages).

Methods (p. 10):

“We also conducted a sensitivity analysis excluding molar pregnancies from the definition of miscarriage.”

Results (p. 12):

“The sensitivity analysis excluding molar pregnancies from the definition of miscarriage showed similar results as the main analysis (S2 Fig.).”

**Comment 3: Selection of chronic diseases: On what basis was chronic diseases included in the analysis - why was Hashimoto thyroiditis not included? Known to be the most common autoimmune manifestation in women of reproductive age and previously found to be associated with the risk of pregnancy loss.**

Response comment 3: In response to this comment, we have included autoimmune thyroiditis as an outcome (ICD-10 code E06.3). Please see revised figures and tables. It is not possible to separate out Hashimoto thyroiditis within the ICD-10 coding system. Autoimmune thyroiditis is coded as E06.3 together with other types of autoimmune thyroiditis.

**Comment 4: Validation of diagnosis: Please include information of diagnosis validation of the included diagnoses. It is surprising that only 1% of the included women suffer from PCOS. This is the most common endocrine disorder in reproductive aged women and supposed to be as high as 5-10%.**

Response comment 4: The diagnoses of chronic diseases in the patient and general practitioner databases have not been systematically validated. However, validations studies are available for a few of the studied conditions. We have incorporated the following sentences into the discussion.

Discussion (p. 15):

“We might have underestimated the number of women with chronic diseases based on information from the registries. This could have resulted in an attenuation of the observed associations due to the presence of chronic diseases also among some women defined as disease free. However, the registration of chronic diseases in in the registries have been shown to have a high validity ^1 2^.”

**Comment 5: Why are numbers of pregnancy losses (the core of this manuscript) not mentioned in Table 1 as it is in supplementary table 2?**

Response comment 5: In response to this comment, we have included the number of miscarriages among women according to underlying chronic diseases in Table 1.

References

1. Benjaminsen E, Myhr KM, Grytten N, et al. Validation of the multiple sclerosis diagnosis in the Norwegian Patient Registry. *Brain Behav* 2019;9:e01422. doi: 10.1002/brb3.1422

2. Bakken IJ, Ariansen AMS, Knudsen GP, et al. The Norwegian Patient Registry and the Norwegian Registry for Primary Health Care: Research potential of two nationwide health-care registries. *Scand J Public Health* 2020;48:49-55. doi: 10.1177/1403494819859737
