# Supplementary material for: Risk of miscarriage in women with chronic diseases in Norway: A registry linkage study
Source: PLoS Med. 2021 May 10;18(5):e1003603. doi: 10.1371/journal.pmed.1003603 (PMC8143388; doi:10.1371/journal.pmed.1003603)
Supplement: Attachment — Submitted filename: Response_letter.docx [file pmed.1003603.s013.docx]

Dear Dr. Arikainen,

We are grateful for the opportunity to publish our manuscript in PLoS Medicine. Please see our responses to the editorial comments and the comment from the reviewer below.

Please accept our sincerely apologies for not responding to one of the comments from reviewer number 3. This was an unfortunate oversight on our part. We hope that our response to this comment below is sufficient.

We refer to the communication we have had with the journal about using miscarriage to define our outcome and not pregnancy loss. While the reviewer is correct about the strict definition of miscarriage, it is common in registry studies to rely on less rigorous definitions of outcomes. The outcome we are studying was recorded by clinicians as either a miscarriage or a pregnancy that did not survive to 16 weeks. Please note that we excluded ectopic pregnancies, so our definition is restricted to uterine pregnancies. This has now been clarified in the methods. We therefore prefer not to use the term “pregnancy loss“ because that could apply to any loss from implantation to term delivery, which is much less specific and could confuse readers. We are grateful for your understanding. As requested, we have also mentioned the limitations of using registries to define miscarriages.

Sincerely,

Maria C. Magnus

Researcher

Centre for Fertility and Health

Norwegian Institute of Public Health

P.O. Box 222 Skøyen

0231 Oslo

Norway

------------------------------------------------------------

Requests from Editors:

**Comment 1.	Please address the reviewer #3’s comment on correct use of terminology – this has also been requested by the Academic Editor. Further consideration of your manuscript is contingent on this being addressed.**

Response comment 1. Please see our comment above. We also refer to our communication with the journal where it was confirmed that the editor allowed us to continue using the term miscarriage for our outcome of interest. We have noted the fact that we excluded ectopic pregnancies in the methods, and we have mentioned the limitations of defining miscarriage outcomes based on registries.

**Comment 2.	Title: Please amend to: “Risk of pregnancy loss in women with chronic diseases in Norway: A registry linkage study”**

Response comment 2. We have left the title as it is due to the fact that we are still using the term miscarriage to define our outcome.

**Comment 3.	Short title: Please amend to: “Chronic diseases and pregnancy loss”**

Response comment 3. We have left the short title as it is due to the fact that we are still using the term miscarriage to define our outcome.

**Comment 4.	Please include line numbers in the margin throughout.**

Response comment 4. We have inserted line numbers as requested.

**Comment 5.	Abstract:**

**a.	Please remove “prospectively” here and throughout, since your analysis itself is retrospective.**

Response comment 5a. We have removed the word prospective/prospectively throughout the manuscript.

**b.	Please remove “retrospective” when describing the study here and throughout, since “registry-based” already implies a retrospective analysis.**

Response comment 5b. We have removed the work retrospective throughout the manuscript.

**c.	Please include p values for your comparisons, alongside 95% CIs.**

Response comment 5c. We have included p-values as requested.

**d.	At the end of the ‘Methods and findings’ subsection, please state more clearly: “Limitations of this study were…”. You could also mention the rareness of some conditions as a further limitation, eg. Cushing’s syndrome.**

Response comment 5d. We have added this limitation to the discussion as requested.

**e.	Conclusions: Please begin with “In this registry study, we found that…”**

Response comment 5e. We have amended the conclusion as described.

**Comment 6.	Page 3: Delete keywords.**

Response comment 6. The keywords have now been deleted.

**Comment 7.	Author Summary: Amend to: “To our knowledge, none of the existing studies have…”**

Response comment 7. We have amended the author summary as described.

**Comment 8.	Please remove spaces from within citations, eg: “…environmental factors [5,6].”**

Response comment 8. We have deleted the spaces from within the citations.

Comment 9.	Results: Please include p values for your comparisons, alongside 95% CIs.

Response comment 9. We have added p-values to the results.

**Comment 10.	Discussion: Please amend to: “Our study is, to our knowledge, unique in its…”**

Response comment 10. We have amended this sentence in the discussion as asked for.

**Comment 11.	Pages 17-18: Please remove the Author Contributions, Data Availability Statement, Funding, and Competing interests sections – these are taken from the online submission form.**

Response comment 11. We have removed these sections from the manuscript file since they are available in the online submission form.

**Comment 12.	References:**

**a.	Please remove city and country from journal names, eg. “(London, England)”.**

**b.	Please delete this from reference 32: “at** [**www.icmje.org/coi_disclosure.pdf**](http://www.icmje.org/coi_disclosure.pdf) **and declare: no support from any organisation for the submitted work; no financial relationships with any organisations that might have an interest in the submitted work in the previous three years; no other relationships or activities that could appear to have influenced the submitted work.”**

**c.	Please delete this from reference 36: “Copyright 2018, The Author(s).”**

Response comment 12. We have made these corrections to the references.

**Comment 13.	Please add the following statement, or similar, to the Methods: "This study is reported as per the Strengthening the Reporting of Observational Studies in Epidemiology (STROBE) and REporting of studies Conducted using Observational Routinely-collected Data (RECORD) guidelines (S1 Checklist)." (and rename the file accordingly)**

Response comment 13. We have added this sentence to the methods as requested.

**Comment 14.	When completing the STROBE/RECORD checklist, please use section and paragraph numbers, rather than page numbers.**

Response comment 14. We have attached an updated STROBE checklist.

---

**Comments from Reviewers:**

**Reviewer #1: The authors have addressed my concerns and I now recommend publication**

**Peter Flom**

Response reviewer #1. We thank you for your time spent reviewing our manuscript.

**Reviewer #3: I am highly surprised that this experienced and respected author group in their rebuttal to PLOS MEDICINE have not responded to a reviewer comment and therefore without any notice keep using miscarriage. To use miscarriage, they should be certain that the pregnancy had been confirmed intrauterine. In practical terms had been scanned, or villi had been identified or the fetus had been visible in the bleeding or the pregnancy material. Clinical researchers changed the terminology to pregnancy loss several years ago unless certain that the losses are intrauterine. I think women pregnant with a gestational age <12 and no prior ultrasound scans with a large bleeding prior to or on their way to hospital who present with an empty uterus at ultrasound and no evaluation of the bleeding will be included in this study.**

Response reviewer #3. We sincerely apologize for not responding to this comment in the initial response letter. This was an unfortunate oversight on our part.

While the reviewer is correct about the strict definition of miscarriage, it is common in registry studies to rely on less rigorous definitions of outcomes. The outcome we are studying was recorded by clinicians as either a miscarriage or a pregnancy that did not survive to 16 weeks. Please note that we excluded ectopic pregnancies, so our definition is restricted to uterine pregnancies. This has now been clarified in the methods. We therefore prefer not to use the term “pregnancy loss“ because that could apply to any loss from implantation to term delivery, which is much less specific and could confuse readers. We are grateful for your understanding. We have also mentioned the limitations of using registries to define miscarriages.

Methods (p. 6):

“Ectopic pregnancies were excluded from the study.”

Discussion (p. 16):

“We also acknowledge that defining pregnancy outcomes based on administrative codes in the registries might have caused misclassification due to our inability to confirm the outcome by clinical examinations.”
