# Supplementary material for: Risk of miscarriage in women with chronic diseases in Norway: A registry linkage study
Source: PLoS Med. 2021 May 10;18(5):e1003603. doi: 10.1371/journal.pmed.1003603 (PMC8143388; doi:10.1371/journal.pmed.1003603)
Supplement: Attachment — Submitted filename: Response_Letter.docx [file pmed.1003603.s014.docx]

Requests from Editors:

**Comment 1: Please amend your data statement to: "Study data are available on application via helsedata.no, subject to the necessary ethics approvals." or similar.**

Response comment 1: We have amended the data statement accordingly.

**Comment 2: Please make that "risk of miscarriage" at line 57.**

Response comment 2: Thank you for spotting this error. We have corrected the sentence as indicated.

**Comment 3: Please state early in the Methods section whether the study had a protocol or prespecified analysis plan. Please highlight analyses that were not prespecified.**

Response comment 3: We have included the following sentence in the methods.

Methods (p. 6):

“The study was conducted according to a pre-specified analysis plan. The only deviation from this plan was that we not only evaluated the risk of miscarriage according to the individual chronic diseases, but also according to subgroups which might share underlying disease pathways.”

**Comment 4: Our Academic Editor has requested that you deal with referee 3's comment explicitly in your discussion section. We ask that you do this by amending the wording at line 322, and suggest: "Our study had important limitations. We acknowledge that the strict definition of miscarriage refers to uterine pregnancies. In our registry study, we defined the outcome as miscarriages or pregnancies that did not survive to 16 weeks; ectopic pregnancies were excluded." or similar.**

Response comment 4: We have included the suggested sentence as requested in the discussion.
